# In Vitro Study of Interleukin-6 when Used at Low Dose and Ultra-Low Dose in Micro-Immunotherapy

**DOI:** 10.3390/life14030375

**Published:** 2024-03-12

**Authors:** Camille Jacques, Flora Marchand, Mathias Chatelais, Adrien Brulefert, Mathieu Riffault, Ilaria Floris

**Affiliations:** 1 Preclinical Research Department, Labo’Life France, Pescalis-Les Magnys, 79320 Moncoutant-sur-Sevre, France; ilaria.floris@labolife.com; 2 ProfileHIT, 7 rue du Buisson, 44680 Sainte-Pazanne, France; flora.marchand@profile-hit.com (F.M.); mathias.chatelais@profile-hit.com (M.C.); 3 QIMA Life Sciences, 1 bis rue des Plantes–CS 50011–86160, 44680 Gençay, France; adrien.brulefert@qima.com; 4 Atlantic Bone Screen (ABS), 3 rue Aronnax, 44800 Saint-Herblain, France; mathieu.riffault@atlantic-bone-screen.com

**Keywords:** micro-immunotherapy, low doses, ultra-low doses, interleukin-6, immune cells models, immune responses modulation, allergy

## Abstract

As one of the major cytokines implicated in the orchestration of immune responses, interleukin 6 (IL-6) can either act as a pro- or an anti-inflammatory factor, depending on the micro-environment. In micro-immunotherapy (MI) medicines, IL-6 is employed at low doses (LD) and ultra-low doses (ULD), expressed in centesimal Hahnemannian (CH), and used alone or in combination with other immune regulators to modulate patients’ immune responses. The present study focused on assessing the in vitro immune-modulatory effects of two IL-6-containing MI products: (i) the unitary IL-6 (4 CH) and (ii) the complex MI-medicine (MIM) 2LALERG^®^, which includes IL-6 (17 CH) in association with other actives in its formulation. Our results showed that IL-6 (4 CH) activated granulocytes under basal conditions, and natural killer cells in the presence of an anti-CD3 signal, as assessed by their CD69 expression. In addition, IL-6 (4 CH) balanced the macrophages’ differentiation toward a M2a profile. On the other hand, the tested 2LALERG^®^ capsule inhibited the histamine degranulation of rats’ peritoneal mast cells and reduced the release of IL-6 itself in inflamed human macrophages. Altogether, these data provide novel pieces of evidence on the double-edged potential of the LD and ULD of IL-6 in immune responses modulation, when employed in MI.

## 1. Introduction

Interleukin-6 (IL-6) is a pleiotropic cytokine known for its involvement in various physiological processes such as hematopoiesis and the coordination of innate/adaptive immune responses, for instance, as illustrated by its participation in B-cells proliferation, plasma cells differentiation, inflammation, and allergy manifestations. The important role played by this small glycoprotein in inflammation and immune reactions could be attested by its immediate and transient expression and secretion, in response to environmental stress factors such as infections and tissue injuries, for example [1]. The importance of IL-6 in host immune defenses is widely investigated. It is well-known that this signaling molecule is involved in practically all the processes of immunity: Th1 and Th2 proliferation, B-cells survival, expansion and maturation, neutrophil infiltration at sites of infection, and many others [2].

From a mechanistic standpoint, IL-6 signalization involves the specific IL-6 membrane-bound receptor (IL-6R) and the common glycoprotein β-receptor 130 kDa (gp130), which results in the activation of the Janus kinase (JAK)–signal transducer and activator of transcription (STAT) pathway [3]. Usually, the activation of STAT3 is triggered through the phosphorylation of an essential tyrosine residue (Tyr 705), leading to the formation of STAT3 dimers due to the interactions between the phospho-tyrosine and SH2 domains [4]. Although various tyrosine kinases, including EGFR, Src, and ERK, have been identified as intracellular stimulators of STAT3 activity, the phosphorylation at tyrosine 705, which is a critical step for STAT3 activation, is predominantly governed by the JAK family, with JAK1 playing a crucial regulatory role [5].

The final step of this cascade is the dimerization of the phosphorylated STATs and their translocation into the nucleus, resulting in the transcription of the IL-6 target genes. Interestingly, while gp130 is ubiquitous, IL-6R is predominantly found in various cell types, such as hepatocytes, muscle cells, and epithelial cells. Additionally, it is also present in various immune cell populations, encompassing subsets of T- and B-cells, monocytes, macrophages, and megakaryocytes [6,7] However, an alternative trans-signaling pathway has been highlighted, the latter relying on a soluble IL-6R (sIL-6R), generated after the cleavage of the membrane-bound IL-6R by the metalloprotease a disintegrin and metalloprotease 17 (ADAM17) [8]. As the IL-6/sIL-6R can still bind gp130, this trans-signaling pathway finally allows all cell types to be responsive to IL-6.

Investigations aimed at assessing the physiological levels of IL-6 showed that its median plasmatic concentration varies between 1.6 and 3.1 pg/mL [9,10]. Nowadays, IL-6 is considered a general marker of immune system activation; it is produced at the site of inflammation, but it has pleiotropic activities, both pro- or anti-inflammatory, depending on the context. It has mainly pro-inflammatory features, and its levels were found upregulated in patients suffering from certain inflammatory diseases. Interestingly, in the context of chronic inflammatory allergic airway diseases, a meta-analysis collecting data from 11 studies conducted from 2017 to 2021, involving 1977 patients with asthma and 1591 healthy individuals, reported that the IL-6 levels found in the serum from asthmatic patients were higher than in non-asthmatic individuals, especially in pediatric patients and in patients with exacerbation [11]. Furthermore, another study revealed that, while the levels of IL-1β and tumor necrosis factor-α (TNF-α) did not significantly differ in induced sputum recovered from mild–moderate asthmatic patients and from healthy controls, the IL-6 levels were more elevated in the first group [12]. These findings indicate the potential role of IL-6 as a biomarker, which could serve as an auxiliary indicator to help make a diagnosis of asthma. 

Considering the pro-inflammatory role of this cytokine in some specific pathological contexts, inhibiting/down-regulating IL-6-mediated pathways could offer promising benefits for patients, and that is why IL-6-targeted therapies have been developed over the last few years and appear as interesting options in several immune-related diseases or even in cancers [13]. Monoclonal antibodies against either IL-6 or IL-6R/sIL-6R, such as siltuximab and tocilizumab, for instance, are investigated in clinics [14,15]. However, on the other side of the spectrum, and depending on the pathological conditions, stimulating the IL-6 signaling could be the way to go. In this attempt, the designer cytokine Hyper-IL-6, which consists of a fusion protein of IL-6 and its α sIL-6R, covalently linked together by a flexible peptide chain, was shown to expand human hematopoietic progenitor cells ex vivo, in a dose-dependent manner [16]. Interestingly, in a mice model of spinal cord injury, the transneuronal delivery of Hyper-IL-6 was recently reported to stimulate the corticospinal tract regeneration and to promote functional recovery after spinal cord crush [17]. In addition, IL-6 has been found to be secreted into the circulation by skeletal muscles during exercise [18], and the plasmatic levels are influenced by the intensity of the exercise [19]. From an immune system standpoint, such cytokine constructs increased neutrophils and macrophages infiltration [20], triggered CD8^+^-T-cells/natural killer (NK) cells-mediated responses [21], and promoted the differentiation of functional dendritic cells [22]. The overall body of knowledge about the role of this cytokine in metabolism and immune responses are important sources of rationales for future applications in clinics. 

Moreover, the pleiotropic function of IL-6 thus offers the opportunity to exploit this cytokine in therapy by either inhibiting or promoting its biological effects, depending on the pathological context. Precisely, such strategies have been employed in micro-immunotherapy (MI), through the inclusion of this cytokine into MI preparations, either at low doses (LD) or ultra-low doses (ULD). Indeed, MI-medicines (MIMs) consist of homeopathic medicinal products, which include signaling molecules, such as cytokines, immune factors, and nucleic acids in their formulations [23,24,25,26]. They all employ small amounts of these active ingredients, expressed as centesimal Hahnemannian (CH) dilutions, impregnated onto sugar pillules, also called globules, for an oromucosal administration. MIMs can either be made of only one active ingredient and are then referred to as unitary MIMs, or they can encompass several ingredients, and are, in this case, qualified as complex MIMs. Another specificity of complex MIMs resides in their sequential administration. Previously published studies have shown the effects of the entire sequence of MIMs, in in vitro models [27] and in in vivo models [25,28,29]. 

The rationale behind the use of the LD/ULD of active ingredients implies that one particular active can either act as an agonist or as an antagonist towards its own physiologically related pathways, depending on the LD or ULD provided to the organism. For instance, in MI, LD (ranging from 3 CH to 5 CH) of a particular active are used in order to display an agonistic effect, resulting in the stimulation of the active’s related biological responses [30,31]. With these considerations in mind, the immune-stimulatory effects of a unitary-MI product containing interferon-γ (IFN-γ) at 4 CH (IFN-γ (4 CH)) have recently been reported [30]. According to those results, IFN-γ (4 CH) showed interesting immune-stimulatory effects in different cell types, opening novel perspectives about the potential of LD-based MIMs in sustaining host immune responses. With the same approach, the present study aims at assessing, for the first time, the in vitro effects of a unitary-MI product containing IL-6, also specifically employed at 4 CH, hereafter referred as IL-6 (4 CH).

On the other hand, in MI, ULD (ranging from 6 CH to 30 CH) of a particular active ingredient are intended to modulate or inhibit its own secretion and biologically-related responses [23,24,25,26,29,32]. Concerning IL-6, previously published research investigated the effects of MI formulations characterized by the presence of ULD of this cytokine in association with other active substances [28,29,32]. In particular, one of these studies pointed to the in vivo effects of the complete sequential administration of the complex-MIM 2LALERG^®^, which contains IL-6 at 17 CH, in reducing the inflammation of the lungs as well as the circulating levels of some T helper cell type 2 (Th2)-related cytokines and immunoglobulin (Ig) E in a mice model of birch pollen-induced allergic inflammation [28]. Furthermore, the rationale behind the IL-6-inhibition strategy through MI, within the context of the atopic march process, has also been previously discussed [33]. In line with this body of literature, the current manuscript is thus also interested in advancing on the preclinical research about the IL-6-ULD-containing MIM 2LALERG^®^. 

Thereby, the results presented here are divided in two different parts: (i) the first one aims at providing more fundamental data about the immune-related effects of IL-6 at the LD of 4 CH (unitary-MI product: IL-6 (4 CH)) (Supplementary Figure S1, left panel); and (ii) the second part is dedicated to the assessment of the effects of one capsule belonging to the complex-MIM 2LALERG^®^ (the composition of which can be found in Materials and Methods), which contains IL-6 at the ULD of 17 CH (Supplementary Figure S1, right panel). 

## 2. Materials and Methods

### 2.1. Tested Items and Experimental Controls

The two tested micro-immunotherapy (MI) formulations are homeopathic medicinal products. The unitary-MI formulation of interest is a low doses (LD)-based medicinal product consisting of sucrose–lactose globules impregnated with ethanolic preparations of human recombinant (hr) IL-6 at 4 CH (centesimal Hahnemannian). The complex MI formulation of 2LALERG^®^ is a sequential MIM, and only one capsule of the sequence was tested in the study. This capsule includes four actives, with the following composition: hr-IL-6 (17 CH); hr-IL-10 (17 CH); hr-transforming growth factor β (hr-TGF-β) (5 CH); and pulmo-histaminum (15 CH). It consists of one of the five capsules belonging to the sequential medicine 2LALERG^®^. These MI formulations were manufactured by Labo’Life España, avenida des Raiguer, 7 07330 Consell—Mallorca, Spain, as previously described [30,31], and have been provided for investigational purposes. The vehicle (Veh.) used in the study as a control consists of sucrose–lactose globules impregnated with ethanol alone. Previous publications described how these Veh. are produced to provide suitable controls for preclinical research [23,24,25,30,31]. In all the experiments performed in the current in vitro study, MI globules of (i) IL-6 (4 CH), (ii) 2LALERG^®^, or (iii) Veh. were freshly diluted in 100 mL of cell culture medium to reach the final sucrose–lactose concentration of 11 mM and were employed to treat the blood cells retrieved from healthy donors

### 2.2. Evaluation of Proliferation and Cell Surface Activation Markers Expression of Peripheral Blood Mononuclear Cells Sub-Populations by Flow Cytometry in Basal Conditions

Healthy volunteers were enrolled by the French Blood Bank Center (Etablissement Français du Sang, [EFS], Pays de Loire, Nantes, France, www.efs.sante.fr: URL accessed on 2 February 2024), a public institution under the responsibility of the French Ministry of Health, and informed consent was obtained from all individuals. According to French ethics laws, blood donation is based on voluntary participation, non-remuneration, anonymity, and non-profitability. A declaration regarding the use of blood samples from blood volunteer donors for non-therapeutic research purposes was submitted to the French Ministry of Research, and the agreement number CPDL-PLER-2019 042 was assigned. Thus, all blood samples were approved by the Ethics Committee of the EFS Blood Bank Center with written informed consent obtained for all the donors, in accordance with the Declaration of Helsinki.

Briefly, peripheral blood mononuclear cells (PBMCs; from three healthy donors, corresponding to a 21-year-old woman, a 35-year-old woman, and a 57-year-old man, and referred to as #A, #B, and #C, respectively) were freshly isolated after Ficoll^®^ gradient separation and cultivated in the presence of either IL-6 (4 CH) or Veh. for 48 h under classical culture conditions: Roswell Park Memorial Institute (RPMI) 1640 medium was added with 2% inactivated human serum, 1 mM non-essential amino acids, 1 mM pyruvate, and 10 mM 4-(2-hydroxyethyl)-1-piperazineethanesulphonic acid (HEPES) buffer. Concanavalin A (Con A; 5 µg/mL) was used as a positive control, regarding its stimulatory effects toward the induction of the CD69 expression. Granulocytes and monocytes/macrophages sub-populations were delineated based on the following panels in the proliferation experiments: granulocytes: CD3^low^, CD11b^high^, CD4^low^, CD8^low^, CD19^low^, CD56^low^, CD14^low^, SSC^high^; monocytes/macrophages: CD3^low^, CD11b^high^, CD4^low^, CD8^low^, CD19^low^, CD56^low^, CD14^high^, SSC^low^. Cell count data were expressed as absolute numbers, concerning the number of the collected beads (Precision Count Beads, #424902, BioLegend, San Diego, CA, USA). In the granulocyte’s activation experiment, the CD69 expression was assessed amongst the granulocyte population characterized by the following panel: CD3^low^, CD11b^high^, CD4^low^, CD8^low^, CD19^low^, CD56^low^, SSC^high^. In the monocytes/macrophages’ activation experiment, the HLA-DR expression was assessed amongst the population characterized by the following panel: CD3^low^, CD11b^high^, CD14^high^, SSC^low^. The cells were labeled and analyzed via flow cytometry on a BD FACS Canto II (BD Biosciences, Franklin Lakes, NJ, USA), configuration 4/2/2.

### 2.3. Evaluation of Cell Surface Activation Markers Expression of Peripheral Blood Mononuclear Cells Sub-Populations via Flow Cytometry, under CD3 Pre-Primed Conditions

Freshly isolated PBMCs were cultivated within the same classical culture conditions described in the previous Section 2.2, with 0.5 µg/mL bottom-coated OKT3 antibody added (anti-CD3, BioLegend, ref: #317325). In the activation assessment experiment, the immune cells’ populations were delineated based on the following panels: NK cells: CD3^low^, CD11b^low^, CD4^low^, CD8^low^, CD19^low^, CD56^high^, CD14^low^, SSC^low^; CD8^+^ T-cells: CD3^high^, CD11b^low^, CD4^low^, CD8^high^, CD19^low^, CD56^low^, CD14^low^, SSC^low^. The CD69 expression level was assessed amongst each of the above-mentioned sub-populations.

### 2.4. Macrophage Viability, Cell Surface Marker Expression and Cytokine Secretion Evaluation

A healthy volunteer (28-year-old woman) was enrolled by the Blood Bank Center (Etablissement Français du Sang, EFS, Pays de Loire, France). The blood sample was approved by the Ethics Committee of the EFS Blood Bank Center with written informed consent obtained for all the donors, in accordance with the Declaration of Helsinki. CD14^+^ monocytes were freshly isolated from PBMCs thanks to the Miltenyi kit (ref #130-050-201, Miltenyi Biotec; Bergisch Gladbach, Germany). Briefly, the cells were then seeded at the density of 50,000 cells/well in 96-well plates on Day 0 and were cultivated in RPMI 1640 supplemented with 2% inactivated human serum, 1 mM nonessential amino acids, 1 mM pyruvate, 2 mM L-glutamine, 10 mM HEPES buffer, and 50 ng/mL macrophage colony-stimulating factor (M-CSF). Cells were treated with either IL-6 (4 CH), Veh., IFN-γ 50 ng/mL (M1 macrophage differentiation inducer), IL-4 50 ng/mL (M2a macrophage differentiation inducer), or IL-10 (M2c macrophage differentiation inducer) from Day 1 until Day 7, with both medium and treatments being renewed on Day 3 and Day 5. Th experimental schemes are shown in Figure 3A and Figure 4A. For the macrophage cell surface marker experiment, the cells were harvested, labeled, and analyzed via flow cytometry on Day 7 on a BD FACS Canto II, configuration 4/2/2. The intensity of the staining was measured as a median fluorescence intensity (MFI) value. The cell viability was appraised based on a gating of the NIR-Zombie-positive cells. The M0, M1, M2a, and M2c macrophage populations were discriminated based on their expression of CD14/CD64/CD86 and identified as follows: M0: CD14^dim^, CD16^dim^, CD163^high^, CD64^high^, HLA-DR^high^, CD86^high^, CD200R^low^; M1: CD14^high^, CD16^dim^, CD163^high^, CD64^high^, HLA-DR^high^, CD86^high^, CD200R^low^; M2a: CD14^low^, CD16^low^, CD163^high^, CD64^low^, HLA-DR^high^, CD86^high^, CD200R^high^; M2c: CD14^high^, CD16^high^, CD163^high^, CD64^high^, HLA-DR^high^, CD86^dim^, CD200R^high^. For the cytokine secretion assessment, the cells were stimulated with lipopolysaccharide (LPS) on Day 6 (100 ng/mL) and harvested on Day 7. Supernatants (SNs) were collected after cell centrifugation, and the cytokine measurement was directly performed via ELISA (LEGENDplex^TM^, BioLegend, San Diego, CA, USA) on fresh SNs. The secretion of the following cytokines was analyzed: TNF-α, IL-1RA, IL-12p40, IL-23, TARC, IP10, IL-10, and IFN-γ. Because only one experiment was conducted, no statistical inference has been performed. Regarding the assessment of the cell viability and the membrane-bound markers, data are represented as mean ± standard error of the mean (S.E.M.) of *n* = 6 replicates for the M0 control and *n* = 3 replicates for all the other conditions. In the case of the cytokine secretion analysis, data are represented as mean ± S.E.M. of *n* = 4 replicates for each M0, M1, M2a and M2c condition and *n* = 3 for the Veh. and the IL-6 (4 CH)-treated condition.

### 2.5. Assessment of the IL-6 Secretion in a Model of CD14^+^-Derived Human Macrophages

The blood sample was retrieved from a healthy 67-year-old male volunteer, enrolled by the Blood Bank Center (Etablissement Français du Sang, EFS, Pays de Loire, France). All blood samples were approved by the Ethics Committee of the EFS Blood Bank Center with written informed consent obtained for all the donors, in accordance with the Declaration of Helsinki. CD14^+^ monocytes were isolated through Ficoll gradient centrifugation and positive selection with magnetic beads (MACS^®^, Miltenyi Biotec, Bergisch Gladbach, Germany). The cells were then cultivated for 6 days in 24-well plates in α-minimum essential media (MEM) added with 10% fetal bovine serum (FBS) and 100 ng/mL M-CSF (ref: #216-MC, R&D Systems, Minneapolis, MN, USA). After 6 days, the medium was replaced with fresh α-MEM medium containing 10% FBS and 50 ng/mL M-CSF, with 100 ng/mL LPS (ref: #L4391, Sigma-Aldrich, St. Louis, MO, USA), Veh., or 2LALERG^®^ added or not. All the treatments were conducted in biological triplicates and applied for 24 and 48 h. At the end of the incubation period, SNs were collected, aliquoted, and frozen at −80 °C until the ELISA analysis. The IL-6 secretion assessment was performed thanks to the human IL-6 Quantikine (R&D Systems). The optical density (OD) was read on a spectrophotometer Multiskan Go, with the Skanlt 3.2 software. 

### 2.6. Assessment of the Activation Levels of Rat Mastocytes through Histamine Degranulation 

Animals were housed in accordance with the European Directive (2010/63/EU) on the protection of animals used for scientific purposes and the French Directives concerning the use of laboratory animals (décret 2013-118, 1 February 2013). Peritoneal mast cells isolated from a pool of *n* = 6 female Wistar rats were pre-incubated in test-optimized Earle’s balanced salt solution (EBSS) medium enriched with 0.1% bovine serum albumin (BSA) in 96-well plates during either 15 or 90 min at the final sucrose–lactose concentration of 11 mM. The cells were then stimulated with 10 µg/mL of the 48/80 compound as an inducer of histamine degranulation for 20 min. A 10 mM cromoglycate (Crgly) solution was employed as a reference compound for histamine degranulation inhibition. The experiments were performed in triplicate and are presented in triplicate, except the Veh. in the 15 min incubation condition, in which the number of replicates was *n* = 2. After the incubation period, SNs were collected and immediately subjected to histamine quantization thanks to an enzyme immunoassay (EIA) histamine kit (Beckman Coulter Life Sciences IMMUNOTECH ref: #IM2562, Marseille, France). 

### 2.7. Statistical Analysis

The graphs in all the figures were performed with GraphPad Prism, Version 9.5.1 for Windows (GraphPad Software Inc., San Diego CA USA, updated on 9 February 2023). The authors have followed the recent recommendations of D.L. Vaux that encourage performing descriptive statistics instead of making statistical inferences when the number of independent values is small [34]. Indeed, no statistical inference has been performed to analyze the results of the studies presented here. If the results are derived from only one, two, or three (*n* = 1, or *n* = 2, or *n* = 3) experiment(s), it is always better to include a full data set, plotting data points and letting the readers interpret the data for themselves, rather than drawing statistical inferences, showing *p* values, the standard error of the mean (S.E.M.), or results that are not representative.

## 3. Results

### 3.1. Under Basal Culture Conditions, the Unitary Micro-Immunotherapy Product IL-6 (4 CH) Activates Granulocytes and, to a Lesser Extent, Monocytes/Macrophages

**Figure 1:**
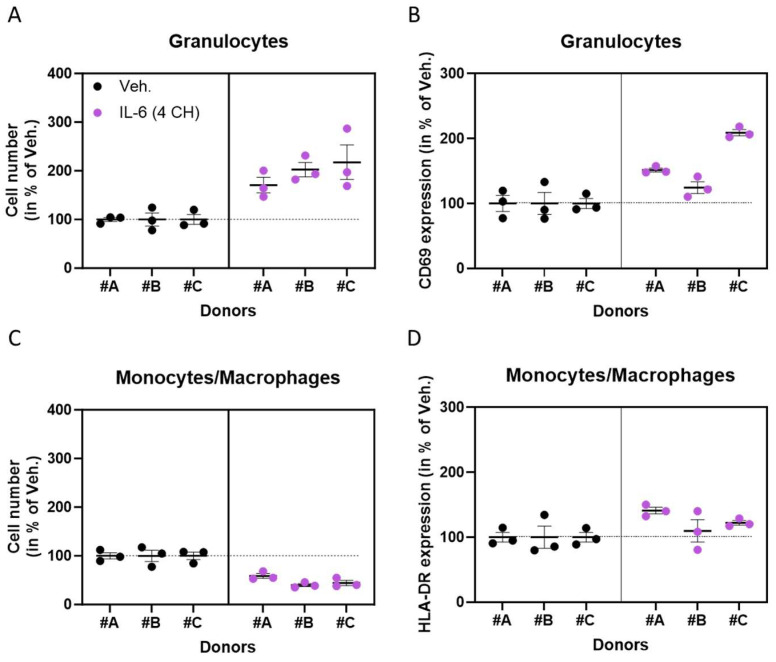
Under basal culture conditions, the unitary micro-immunotherapy (MI) product IL-6 (4 CH) activates granulocytes and, to a lesser extent, monocytes/macrophages. (**A**–**D**) In a basal state, the cell number (**A**,**C**), and the activation status (**B**,**D**) of granulocytes and monocytes/macrophages have been evaluated via flow cytometry, after 48 h of peripheral blood mononuclear cells (PBMCs)’ culture in the presence of either IL-6 (4 CH) or the vehicle (Veh.), isolated from three healthy donors (#A, #B, and #C). The activation of granulocytes was assessed by measuring the levels of CD69, although the activation of monocytes/macrophages was evaluated by monitoring the levels of human leukocyte antigen–DR (HLA-DR). For each donor, the data are presented as the mean ± standard error of the mean (S.E.M.) obtained for *n* = three replicates and are expressed as a percentage of the Veh. (set at 100% for each donor). Each black dot represents one replicate per donor in the Veh. condition, whereas purple dots represent the replicates in the IL-6 (4 CH)-treated condition. The dotted black lines are drawn to highlight the effects of IL-6 (4 CH) in comparison with the Veh. condition.

In order to assess the capabilities of the unitary micro-immunotherapy product IL-6 (4 CH) to influence immune responses, especially the ones related to the innate side of immunity, we first investigated its effect on the granulocytes and monocytes/macrophages sub-populations, cultivated under basal conditions. Peripheral blood mononuclear cells (PBMCs) from three healthy donors were thus isolated and incubated for 48 h with either the vehicle (Veh.) control (which consists of the sucrose–lactose globules alone) or with the unitary-MI product IL-6 (4 CH). A multiparametric immuno-analysis was performed via flow cytometry, assessing the cell proliferation and the expression of two activation markers, CD69 and HLA-DR, within the granulocytes and the monocytes/macrophages, respectively. The granulocytes’ activation was validated by performing an incubation with 5 µg/mL of concanavalin A (Con A) for 48 h and by measuring the expression level of CD69 (Supplementary Figure S2). As shown in Figure 1A,B and Supplementary Table S1, IL-6 (4 CH) increased the number of living cells and the expression of CD69 in the granulocytes from the three tested donors. A different response pattern was obtained in the monocytes/macrophages sub-population, in which IL-6 (4 CH) reduced the cell number, a feature of the reduced capacity of proliferation, while still increasing, even if just slightly, the expression of the HLA-DR marker (Figure 1C,D and Supplementary Table S1). 

Overall, this first set of data seemed to highlight the opposite effects of IL-6 (4 CH) on cell proliferation within the two sub-populations analyzed as the latter seemed to act as a stimulatory factor in granulocytes but as an inhibitory factor in monocytes/macrophages. However, the consistent capability of IL-6 (4 CH) to increase the activation level of granulocytes and monocytes/macrophages could also be featured. 

### 3.2. In the Presence of an Anti-CD3 Signal, the Unitary Micro-Immunotherapy Product IL-6 (4 CH) Acts as a Co-Stimulator for Natural Killer Cells and, to a Lesser Extent, CD8^+^ T-Cells

**Figure 2:**
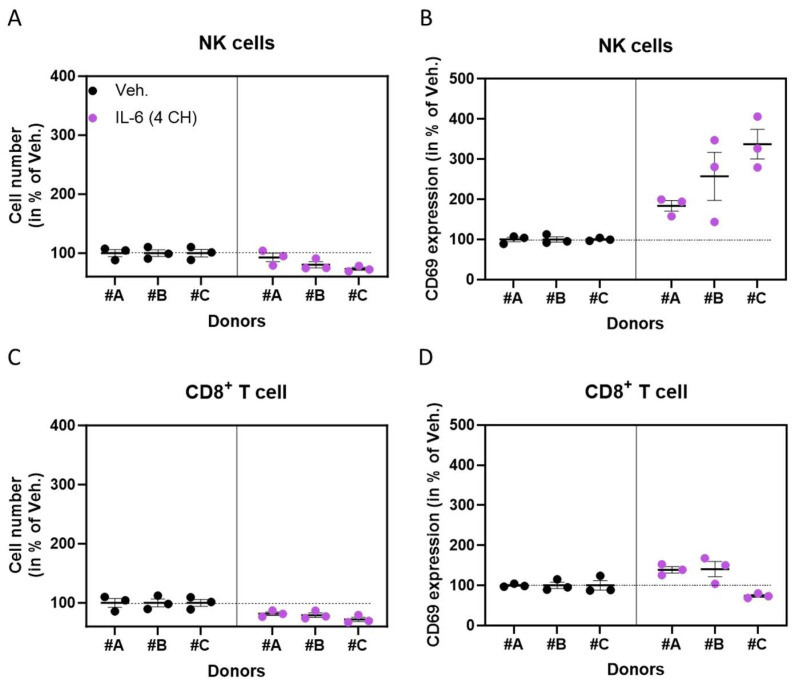
In the presence of an anti-CD3 signal, the unitary micro-immunotherapy (MI) product IL-6 (4 CH) activates natural killer (NK) cells and, to a lesser extent, CD8^+^ T-cells. In the CD3-primed state, the cell number (**A**,**C**) and the activation status (**B**,**D**) of NK cells and CD8^+^ T-cells were evaluated via flow cytometry, after 48 h of peripheral blood mononuclear cells (PBMCs)’ culture in the presence of 0.5 µg/mL of an anti-CD3 and either the vehicle (Veh.) or IL-6 (4 CH). The PBMCs were isolated from three healthy donors (#A, #B, and #C). The activation of the NK cells and the CD8^+^ T-cells was assessed by measuring the expression levels of CD69. For each donor, the data are presented as the mean ± S.E.M. results obtained for *n* = three replicates and are expressed as a percentage of the Veh. (set at 100% for each donor). Each black dot represents one replicate per donor in the Veh. condition, whereas purple dots represent the replicates in the IL-6 (4 CH)-treated condition. The dotted black lines are drawn to highlight the effects of IL-6 (4 CH) in comparison with the Veh. control.

We then wanted to assess the capability of the unitary-MI product IL-6 (4 CH) to act as a co-stimulator for the immune cell populations. For this purpose, the experiment was carried out in the presence of an anti-CD3 to provide a stimulatory signal for the T-cells. The responsiveness of the assessed cells’ sub-populations was evaluated by measuring the CD69 expression after incubation in the presence of an anti-CD3 alone or with 5 µg/mL of Con A for 48 h as a reference treatment for natural killer (NK) cells activation [35]. Once the model was validated by the increased expression of CD69 in both Con A and anti-CD3-treated cells (Supplementary Figure S3), we tested the effects of IL-6 (4 CH) in association with an anti-CD3. In the presence of the anti-CD3 signal and IL-6 (4 CH), both NK cells and CD8^+^ T-cells displayed a change in their proliferative capacity and their CD69 expression pattern in comparison with the Veh. (Figure 2 and Supplementary Table S2). Indeed, in these two cell sub-populations, IL-6 (4 CH) induced a slight reduction in the cell count, observed in the three donors (Figure 2A,C and Supplementary Table S2). Concerning the investigations about the possible role of IL-6 (4 CH) as a co-stimulator in the same immune sub-populations, the overall results showed that it increased their CD69 expression (Figure 2B,D) when compared with the Veh. condition. In particular, the increase in the CD69 expression was stronger in the NK cells compared with the CD8^+^ T-cells. Indeed, in NK cells, the strongest response was observed in donor #C, in which the CD69 increase was of about 340% in comparison with the Veh., while the weakest response, obtained in donor #A, manifested with an increase in the CD69 expression by about 180% compared with the Veh. (Figure 2B). Regarding the CD8^+^ T-cells, while the CD69 expression was increased by about 140% in comparison with the Veh. in donor #A and donor #B, this marker decreased in donor #C (Figure 2D). 

Overall, this second set of data seemed to highlight the capability of IL-6 (4 CH) to outstandingly increase the activation level of NK cells and the one of CD8^+^ T-cells, to a lesser extent, in a CD3-pre-primed state. Having observed a much less pronounced effect on cell proliferation, we prefer to stay cautious with these results. 

### 3.3. The Unitary Micro-Immunotherapy Product IL-6 (4 CH) Modulates the Expression of Membrane-Bound Markers of CD14^+^-Derived Macrophages

**Figure 3:**
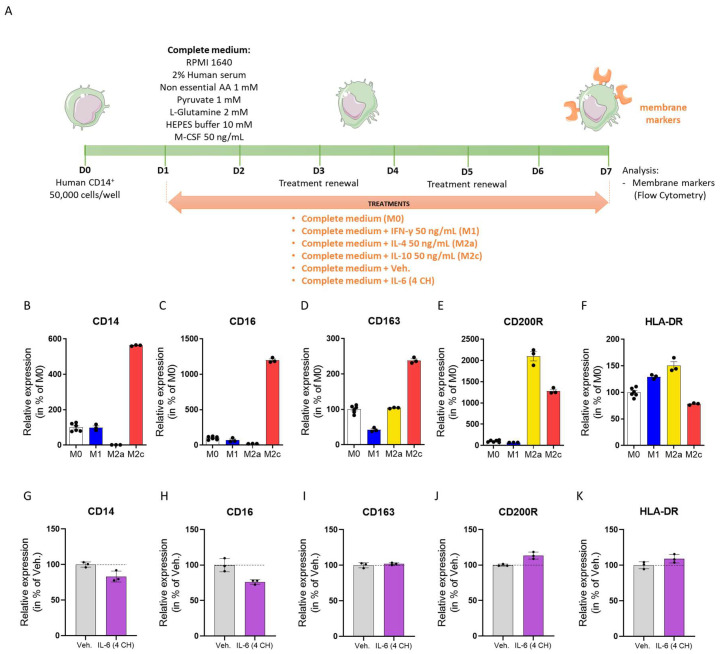
(**A**) The unitary micro-immunotherapy (MI) product IL-6 (4 CH) modulates the expression of membrane-bound markers of CD14^+^-derived macrophages. (**A**) Representative scheme of the experimental protocol. CD14^+^ cells were obtained from peripheral blood mononuclear cells (PBMCs) isolated from one healthy donor and cultivated in a complete medium supplemented with 50 ng/mL macrophage colony-stimulating factor (M-CSF) (M0) for 7 days. The polarization of the CD14^+^ cells into macrophages was conducted through the adjunction of either 50 ng/mL IFN-γ (M1), 50 ng/mL IL-4 (M2a), or 50 ng/mL IL-10 (M2c) at Day 1 (D1) up until Day 7 (D7). In parallel, the Veh. or IL-6 (4 CH) was employed to treat the cells according to the same timeline. Cell surface markers expression was assessed via flow cytometry. AA: amino acids. (**B**–**K**) Effects of IL-6 (4 CH) on the expression of the cell surface markers CD14, CD16, CD163, CD200R, and HLA-DR in the M0 control macrophages (white histograms), M1 (blue histograms), M2a (yellow histograms), and M2c (red histograms) (**B**–**F**), in the Veh. (gray histograms) and the IL-6 (4 CH)-treated cells (purple histograms) (**G**–**K**). Data are represented as mean ± S.E.M. of *n* = six replicates for the M0 control and *n* = three replicates for all the other conditions. For each one of the analyzed markers, the results are expressed in percentage of either the M0 macrophage control condition (**B**–**F**) or in percentage of the Veh. condition (**G**–**K**). The dotted black lines are drawn to highlight the effects of IL-6 (4 CH) in comparison with the Veh. control.

As mentioned in the first section of the results in this study, the unitary-MI product IL-6 (4 CH) could activate the monocyte/macrophage sub-population and specifically increase the expression of CD69. To further investigate the role of the tested item on macrophages, we then checked its effects on the polarization state of human macrophages. We thus evaluated the effects of the unitary-MI product IL-6 (4 CH) on the expression pattern of unpolarized M0 macrophages in order to compare it to M1-, M2a- and M2c-differentiated macrophages. The overall protocol of the experiment is illustrated in Figure 3A, but briefly, primary CD14^+^ cells were isolated from the PBMCs of one healthy human donor, and in order to generate the appropriate experimental controls for macrophage differentiation, the cells were cultivated for seven days in complete medium, which included 50 ng/mL of macrophage colony-stimulating factor (M-CSF), to promote survival and make them differentiate into M0 macrophages. This complete medium was supplemented with either 50 ng/mL IFN-γ (M1), 50 ng/mL IL-4 (M2a), or 50 ng/mL IL-10 (M2c), respectively, for each macrophage control. In parallel, either the Veh. or the unitary product IL-6 (4 CH) were applied on the cells on Day 1 (D1) after CD14^+^ isolation and plating, up until Day 7 (D7), with the medium/treatments being renewed twice, on Day 3 (D3) and Day 5 (D5). The cell viability was not impacted by the macrophages’ differentiation protocol (Supplementary Figure S4A) nor by the Veh. or the IL-6 (4 CH) treatment (Supplementary Figure S4B). The expression of the membrane markers CD14, CD16, CD163, CD200R, and HLA-DR was assessed via flow cytometry in the M0, M1-, M2a-, and M2c-differentiated macrophages, as well as in the Veh. and the IL-6 (4 CH)-treated ones (Figure 3B–K). As shown in Figure 3B,D, while the expression of CD14 and CD16 was found to be reduced in M2a macrophages vs. M0 macrophages, no change in the level of CD163 was noticed between these two macrophages sub-types. Interestingly, a similar expression pattern was observed for these three markers as a consequence of IL-6 (4 CH) treatment, when compared to the Veh. one (Figure 3G–I). Additionally, the increased expression of CD200R was a feature of both the M2a and the M2c macrophages, when compared with the M0 control ones (Figure 3E). In the same manner, IL-6 (4 CH) induced an increase in the expression of CD200R, in comparison with the Veh. condition (Figure 3J). Finally, a slight increase in the expression of HLA-DR was found after IL-6 (4 CH) treatment in comparison with the Veh. (Figure 3K). The latter can be seen as a signature of both the M1 and the M2a macrophages vs. M0 macrophages (Figure 3F). 

Altogether, and under the experimental setting tested here, these results could possibly sustain a pro-M2a differentiation effect of the unitary-MI product IL-6 (4 CH) when compared with the Veh. condition.

### 3.4. The Unitary Micro-Immunotherapy Product IL-6 (4 CH) Modulates the Cytokines Secretion of CD14^+^-Derived Macrophages

**Figure 4:**
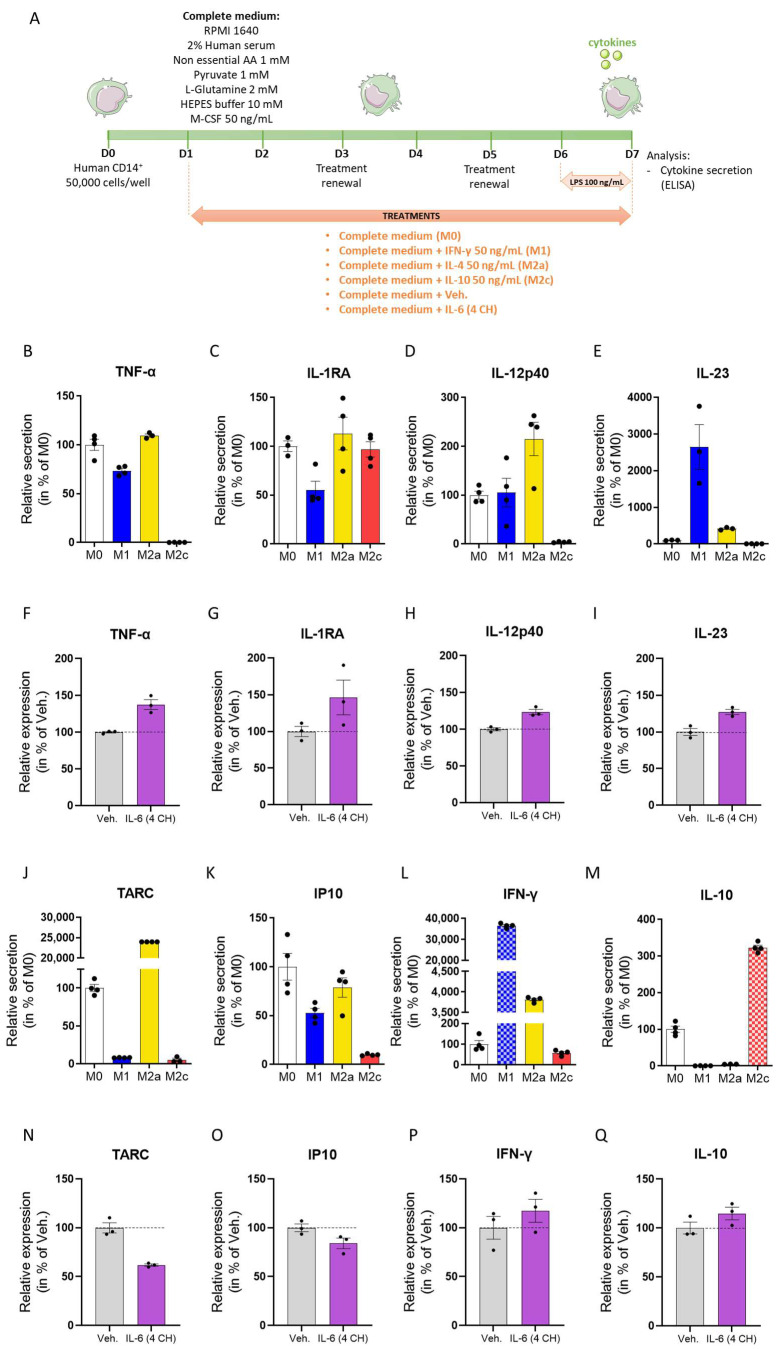
(**A**) The unitary micro-immunotherapy (MI) product IL-6 (4 CH) modulates the cytokine secretion patterns of CD14^+^-derived macrophages in favor of a balanced M1/M2 profile. (**A**) Representative scheme of the experimental protocol. CD14^+^ cells were obtained from peripheral blood mononuclear cells (PBMCs) isolated from one healthy donor and cultivated in a complete medium supplemented with 50 ng/mL macrophage colony-stimulating factor (M-CSF) (M0) for 7 days. The polarization of the CD14^+^ into macrophages was conducted through the adjunction of either 50 ng/mL IFN-γ (M1), 50 ng/mL IL-4 (M2a), or 50 ng/mL IL-10 (M2c) at Day 1 (D1) up until Day 7 (D7). In parallel, the vehicle (Veh.) or IL-6 (4 CH) was employed to treat the cells according to the same timeline. A 24 h lipopolysaccharide (LPS) treatment (100 ng/mL) was applied as an inducer of an inflammatory status. The concentration of cytokines in the cells’ supernatants (SNs) was evaluated via ELISA. AA: amino acids. Effects of IL-6 (4 CH) on the secretion of the cytokines TNF-α, IL-1RA, IL-12p40, IL-23, TARC, IP10, IFN-γ, and IL-10 in the M0 control macrophages, M1, M2a, and M2c (**B**–**E**,**J**–**M**), and in the vehicle (Veh.)- and the IL-6 (4 CH)-treated cells (**F**–**I**,**N**–**Q**). The squared blue and squared red histograms depicted in (**L**) and (**M**), respectively, illustrate the experimental conditions in which IFN-γ and IL-10 were used to induce macrophage polarization. In these conditions, the amount of cytokines quantified via ELISA thus reflects not only the secreted levels in the SNs but also the exogenous amount provided by each respective cell treatment. Data are represented as mean ± S.E.M. of *n* = four replicates for each M0, M1, M2a, and M2c conditions and *n* = three for the Veh.- and the IL-6 (4 CH)-treated conditions. For each one of the analyzed cytokines, the results are expressed in percentage of either the M0 macrophage control condition (**B**–**E**,**J**–**M**) or in percentage of the Veh. condition (**F**–**I**,**N**–**Q**). The dotted black lines are drawn to highlight the effects of IL-6 (4 CH) in comparison with the Veh. control.

In order to complement the previous analysis of the CD14^+^-derived macrophages, the cytokine secretion profile of these cells was also assessed in the same previously described conditions. As depicted in Figure 4A, the cytokine secretion was induced by the adjunction of 100 ng/mL lipopolysaccharide (LPS) to the culture media applied on Day 6 (D6). The cytokine content in the supernatants (SNs) was analyzed via an enzyme-linked immunosorbent assay (ELISA) for the following cytokines: TNF-α, IL-1RA, IL-12p40, IL-23, thymus, and activation-regulated chemokine (TARC), IP10, IFN-γ, and IL-10. As an additional control for the validity of this experiment, the cell viability was also evaluated in these LPS conditions. The latter was not impacted by the inflammatory trigger, neither in the cells undergoing macrophage differentiation (Supplementary Figure S5A), nor in the ones treated with the Veh. or IL-6 (4 CH) (Supplementary Figure S5B). The secretion levels of TNF-α, IL-1RA, and IL-12p40 were all increased in the M2a macrophages compared with the M0 (Figure 4B,D), as well as in the IL-6 (4 CH)-treated macrophages in comparison with the Veh.-treated ones (Figure 4F–H). Interestingly, the increase in the secretion of these three cytokines was solely found in the M2a sub-type and was not a signature of the M1 or the M2c macrophages (Figure 4B,D). In parallel, an increase in the IL-23 production was found in both M1 and M2a macrophages vs. M0 macrophages, as well as in the IL-6 (4 CH) treatment condition vs. Veh. (an approximate 30% increase compared with the Veh. treatment) (Figure 4E,I). On the opposite side of the spectrum, both M1 and M2c macrophages were found to decrease their TARC secretion compared with the M0 ones (Figure 4J), and IL-6 (4 CH) also reduced it by about 40% when compared with the Veh. (Figure 4N). Similarly, the secretion of IP10 was reduced in the three M1-, M2a-, and M2c-differentiated macrophages, as well as in the IL-6 (4 CH)-treated ones (Figure 4K,O). Finally, the secretion levels of IFN-γ and IL-10 were also assessed in the three differentiated macrophage sub-types (Figure 4L,M) and in the IL-6 (4 CH)/Veh.-treated ones (Figure 4P,Q). IL-6 (4 CH) induced an increase in the IFN-γ and in the IL-10 secretion of about 15% compared with the Veh. (Figure 4P,Q). While an increase in the IFN-γ measured in the SNs from M1/M2a macrophages vs. M0 macrophages was observed (Figure 4L), an increase in the IL-10 measured in the SNs from M2c macrophages vs. M0 macrophages was also noticed. However, it is important to keep in mind that the culture medium of the M1 and the M2c macrophages were enriched with IFN-γ and IL-10 in order to induce differentiation. Consequently, the levels measured in those two conditions cannot be solely attributed to the secreted levels. For this reason, the histograms are highlighted with squared blue and squared red colors for IFN-γ and IL-10, respectively, in Figure 4L,M. In conclusion, even if some of the cytokine secretion patterns here seemed to be similar to the ones found in M1 macrophages (IL-12p40, IL-23, TARC, and IP10) and in M2c macrophages (TARC and IP10), the majority of them resembled the one observed in M2a macrophages (TNF-α, IL-1RA, IL-12p40, IL-23, IP10, and IFN-γ). 

Collectively, these data about the cytokine secretion profile found after M0 macrophages treatment with the unitary-MI product IL-6 (4 CH) seemed to be in line with the above-presented observations about the membrane markers (Section 3.3), as both sets of data suggest that this unitary-MI product could orient the polarization of the macrophages toward an M2a profile.

### 3.5. The ULD-IL-6-Containing Capsule of the Complex Micro-Immunotherapy Medicine 2LALERG^®^ Displays an Inhibitory Effect on IL-6 Secretion in a Model of CD14^+^-Derived Human Macrophages

**Figure 5:**
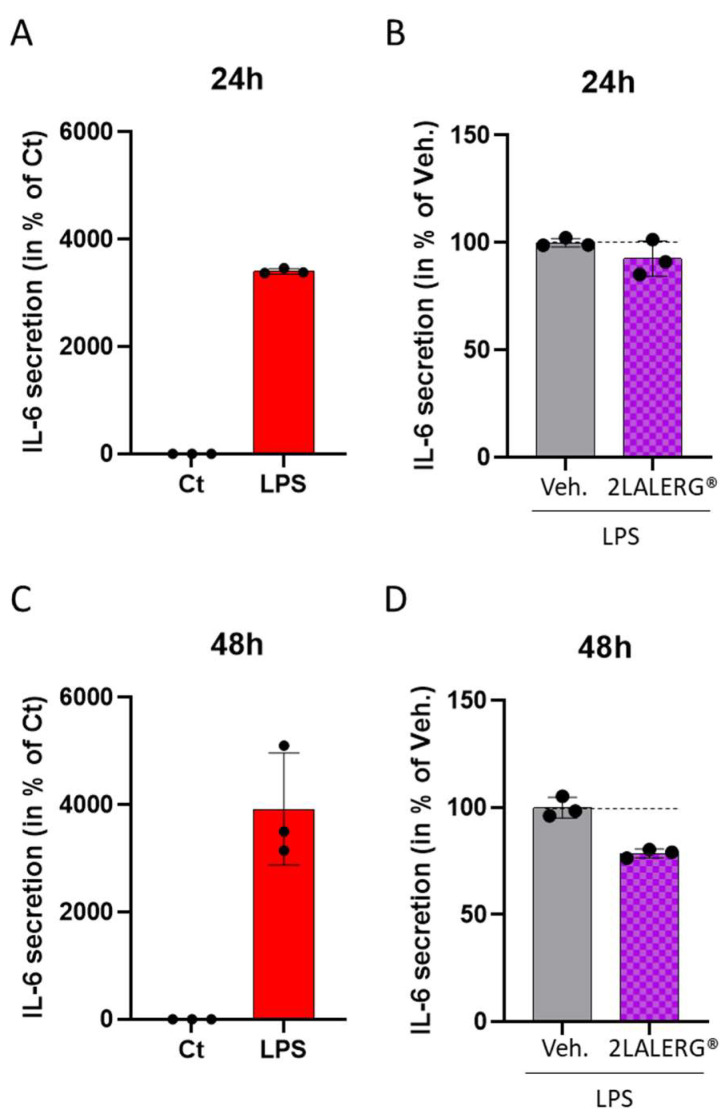
The ultralow dose (ULD)-IL-6-containing capsule of the complex micro-immunotherapy medicine (MIM) 2LALERG^®^ displays an inhibitory effect on IL-6 secretion in a model of human macrophages. CD14^+^ cells from one healthy donor were isolated and differentiated into M0 macrophages for 6 days, before being treated with/without 100 ng/mL lipopolysaccharide (LPS) alone or with 100 ng/mL LPS + either the vehicle (Veh.) or one capsule of 2LALERG^®^ during (i) 24 h (upper panel) or (ii) during 48 h (lower panel). The cytokines secreted in the supernatants (SNs) were collected, and the IL-6 content was analyzed via ELISA. The results are presented after 24 h of LPS treatment alone (**A**) or concomitantly with either the Veh. or 2LALERG^®^ (**B**). The same respective treatment conditions were evaluated after 48 h of treatment (**C**,**D**). Data are represented as mean ± standard deviation (S.D.) of *n* = three replicates for each condition. The results are expressed in percentage of either the non-treated M0 macrophage control condition (Ct) (**A**,**C**) or in percentage of the Veh. condition (**B**,**D**). The dotted black lines are drawn to highlight the effects of 2LALERG^®^ in comparison with the Veh. control.

The second part of this study focused on assessing the effect of IL-6 when employed at ULD in a complex MIM. Thus, one capsule of the complex-MIM 2LALERG^®^, as it contains IL-6 (17 CH) in its formulation, was tested in an in vitro model of inflammation. Especially, its capacity to affect the secretion of IL-6 was evaluated here. Human CD14^+^ monocytes were thus isolated from one healthy donor and were cultivated for 6 days in a medium enriched with M-CSF to make them differentiate into M0 macrophages. To provide a pro-inflammatory stimulus, the cells were incubated with 100 ng/mL LPS for either 24 h or 48 h and were concomitantly treated with either the Veh. or the 2LALERG^®^ capsule. At the end of the incubation period, SNs were collected, and the IL-6 content was evaluated via ELISA in each treatment condition. As illustrated in Figure 5A,C, the macrophages were able to drastically increase their LPS-stimulated-IL-6 secretion, when compared with the unstimulated controls, at the end of the two tested incubation periods. Interestingly, in the presence of LPS, the tested 2LALERG^®^ capsule induced a reduction in the cells’ IL-6 secretion of about 8% after 24 h of treatment compared with the Veh. condition (Figure 5B) and an inhibition of about 20% after the completion of a 48 h treatment (Figure 5D). 

In conclusion of this set of experiments, the tested capsule of 2LALERG^®^ seemed to reduce the LPS-induced IL-6 secretion of human macrophages. While specific assessments with the only unitary MIM are necessary to validate our hypothesis, these data could be explained by the presence of IL-6 (17 CH) in the formulation of this medicine. 

### 3.6. The ULD-IL-6-Containing Capsule of the Complex Micro-Immunotherapy Medicine 2LALERG^®^ Displays an Inhibitory Effect towards Rat Mast Cells’ Histamine Degranulation

Regarding the fact that IL-6 is a well-known stimulator of mast cells’ histamine degranulation, we wanted to address the question if this particular feature could be affected by the composition of the complex-MIM 2LALERG^®^, which includes a ULD of IL-6. In addition, the tested capsule also contains a ULD of pulmo-histaminum, specifically at 15 CH.

**Figure 6:**
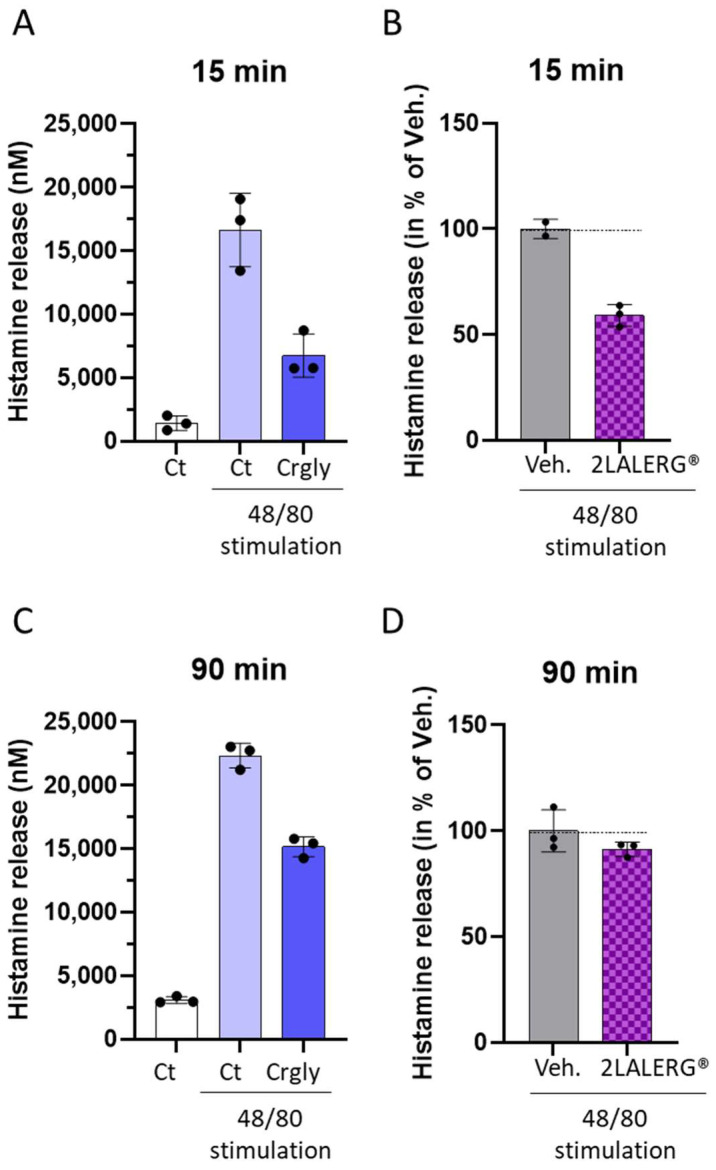
The tested capsule of the complex micro-immunotherapy medicine (MIM) 2LALERG^®^ inhibits histamine degranulation in rats’ mast cells. Rat’s peritoneal mast cells were retrieved from a pool of *n* = six female rats, pre-incubated for 15 min with/without either cromoglycate (Crgly) (**A**), vehicle (Veh.) or 2LALERG^®^ (**B**), before 10 µg/mL of 48/80 compound was added for the next 20 min as an inducer of histamine degranulation. The histamine content in the supernatants (SNs) was measured via enzyme immunoassay (EIA) assay for each condition. The same protocol was followed by a pre-incubation of 90 min with/without either Crgly (**C**), Veh., or 2LALERG^®^ (**D**). Data are represented as mean ± S.D. for each condition. The results are expressed in nM of histamine in the mast cells used for the control conditions (Ct) (**A**,**C**) or in percentage of the Veh. condition (**B**,**D**). The dotted black lines are drawn to highlight the effects of 2LALERG^®^ in comparison with the Veh. control.

In this experiment, peritoneal mast cells were isolated from Wistar rats and were pre-incubated for either 15 min or 90 min with the tested capsule. The cells were then stimulated with 10 µg/mL of the 48/80 compound (an inducer of histamine degranulation) during the next 20 min, and the histamine content in the SNs was measured via an enzyme immunoassay (EIA). A solution of 10 mM cromoglycate (Crgly) was used as a positive control towards histamine degranulation inhibition and was also pre-incubated for either 15 or 90 min. As illustrated in Figure 6A, the stimulation with the 48/80 compound induced a drastic increase in the histamine release, which the Crgly was able to repress by about 60% after only 15 min of incubation. Interestingly, the 2LALERG^®^ capsule displayed a repressive effect toward histamine release by about 40% compared with the Veh., under the same incubation period (Figure 6B). The same trends were found after 90 min of incubation, but the effects of both Crgly and the 2LALERG^®^ capsule were less powerful toward histamine inhibition (about 30% of inhibition for Crgly [Figure 6C] and about 10% of inhibition for 2LALERG^®^ capsule, compared with their respective controls [Figure 6D]). 

Altogether, this final body of data suggested that the tested capsule of 2LALERG^®^ may have had an inhibitory effect toward the 48/80-induced histamine degranulation, after short incubation periods, in a model of rat mast cells, possibly mediated by the capacity of this MIM to reduce the secretion of IL-6, as mentioned in the above paragraph of results.

## 4. Discussion

### 4.1. General Description of the Main Results of the Study

Our results seemed to show that the unitary-MI product IL-6 (4 CH) activated the granulocytes and the monocytes/macrophages sub-populations isolated from the peripheral blood mononuclear cells (PBMCs) of three healthy donors, when these cells were cultivated under basal culture conditions, as assessed by their expression levels of CD69 and human leukocyte antigen–DR (HLA-DR). Moreover, IL-6 (4 CH) also may have acted as a co-stimulator for NK cells and CD8^+^ T-cells, in the presence of an antibody against CD3, thus possibly acting as an immune stimulatory factor. In a model of CD14^+^-derived M0 macrophages, IL-6 (4 CH) seemed to induce a modulation of the membrane markers CD14, CD16, CD163, CD200R, and HLA-DR, which phenocopied the expression pattern found in M2a-differentiated macrophages. In addition, it also modulated the lipopolysaccharide (LPS)-induced cytokine secretion of M0 macrophages toward a profile that looked quite similar to the one found in M2a, regarding the expression levels of TNF-α, interleukin-1 receptor antagonist (IL-1RA), IL-12p40, IL-23, interferon γ-induced protein 10 (IP10), and IFN-γ. 

On the other hand, our results also provide data on the use of a complex-MIM employing IL-6 at the ULD of 17 CH, combined with other active ingredients into one capsule of the complex-MIM 2LALERG^®^. In a model of CD14^+^-derived macrophages inflamed with LPS, the tested formulation seemed to reduce the secreted levels of IL-6 and inhibited the histamine degranulation of rats’ peritoneal mast cells. 

Taken together, and even if still relatively preliminary, these data provide, for the first time, novel pieces of evidence on the double-edged potential of LD and ULD of IL-6, either tested under the form of the MI unitary product IL-6 (4 CH) or included into the complex MIM’s formulation of 2LALERG^®^, in modulating the immune cells’ responses toward an activation or an inhibition, respectively. Although more preclinical and clinical research is needed in this area, these results shed light on the interesting therapeutic potential of the delivery of LD and ULD of IL-6 to the organism, through MI.

### 4.2. Discussion about the Effects of Low Doses of IL-6, Studied through the Unitary-MI Product IL-6 (4 CH)

In the first part of this study, we assessed the effect of a unitary micro-immunotherapy (MI) preparation of IL-6, employed at the low dose (LD) of 4 CH (centesimal Hahnemannian). Knowing how important this cytokine is in the context of host immune defenses [36], the approach followed here was intended to assess its effects on general immune cell proliferation and activation. 

Here, the results thus suggested that IL-6 (4 CH) could enhance the capacity of the proliferation of granulocytes or at least that it may have contributed to an increase in the number of these cells as well as their activation, as monitored by the augmented expression of the membrane marker CD69 in these cells (Figure 1A,B). Regarding this, previous observations from a clinical study performed on healthy young volunteers who received an infusion of IL-6 reported an association between the transient increase in plasmatic IL-6 and the number of neutrophils [37]. In addition, a slight increase in the expression of CD69 was also reported by Atzeni et al., after treating human neutrophils with 100 U/mL IL-6 for 18 h [38]. It is interesting to mention that numerous other markers related to immune cell activity can be analyzed, and in this context, Borish et al., also reported the role of IL-6 as an immune stimulator toward neutrophil activation as they found that a recombinant form of this cytokine has stimulated the secretion of lysozyme, lactoferrin, and β-glucuronidase [39]. Even if the direct role played by IL-6 on granulocytes still needs to be investigated, it has recently been suggested in a study aimed at estimating the number of IL-6 needed to activate a target cell that granulocytes should have forty-eight IL-6-binding sites per cell [40]. This same study also revealed that only four molecules of IL-6 per cell were significantly able to induce a cellular response, pointing out the extreme sensitivity of immune cells to cytokines. 

Going back to our findings, our results about monocytes/macrophages seemed to show that, when compared to the vehicle (Veh.)-treated peripheral blood mononuclear cells (PBMCs), a 48 h treatment with IL-6 (4 CH) may induce a decrease of about 50% in the number of these cells, when averaged on the three analyzed donors (Figure 1C). These results are in correlation with a study that has put forward the reversible inhibitory effects of IL-6 on the proliferation of both progenitors and differentiated macrophages [41]. In the same monocytes/macrophages population, the treatment induced an increased expression of HLA-DR by an average of approximately 25% (Figure 1D). While the mechanism behind this phenomenon should be investigated, the overall results seemed to highlight the existence of a positive correlation between the levels of IL-6 and the expression levels of HLA-DR. 

Interestingly, the results that we obtained about HLA-DR expression in monocytes/macrophages (Figure 1D) are consistent with the observation that we have also made in primary CD14^+^-derived macrophages (Figure 3K), in which a 6-day-treatment with IL-6 (4 CH) could also, even if slightly, increase the expression of HLA-DR in those cells (by about 10%). 

As IL-6 is often found as a cytokine that is up-regulated during the course of acute and chronic infections, in which adaptive immunity plays a critical role [42], and regarding the recent advances in documenting the biological activity of IL-6 as a shift from innate to acquired immunity [43], we wanted to complement the first set of data with more information about the effect of the unitary micro-immunotherapy (MI) product IL-6 (4 CH) in the context of a pre-activated adaptive immunity. Thus, such pre-activation was mimicked by a CD3-primed environment, in which, we found that IL-6 (4 CH) may display a strong increase in the CD69 expression in natural killer (NK) cells (Figure 2B) and a slight inhibitory effect toward their proliferation (Figure 2A). The role played by IL-6 in NK cells is still obscure and, up to now, contradictory data exist. For instance, a study investigating the effect of 30 ng/mL of this cytokine reported that it increased the NK cells differentiation process at early stages while decreasing the cytolytic activity of mature NK cells [44]. Nonetheless, concerning the CD8^+^ T-cells, our results also seemed to highlight a response pattern that appears similar to the one observed for the NK cells as a slight reduction in the cell proliferation was also found, in a CD3-primed context, as a consequence of an IL-6 (4 CH) treatment, in comparison with the Veh. (Figure 2C).

We also observed an increase of about 40% in CD69 expression in two out of the three tested donors (Figure 2D; donors #A and #B). As sustained by Krowka et al. [45], it is worth mentioning that, even though the expression of CD69 is considered a marker of early activation, it does not necessarily reflect the proliferation levels of the cells. Indeed, in their work on human immunodeficiency virus-positive and -negative T-cells responses, these authors reported that, while the CD69 expression could be detected in response to stimuli such as phorbol myristate acetate, which activated T-cells, this stimulation did not drive all the way to proliferation. Furthermore, even if very few data are available on the relationship between IL-6 and CD69 in pre-activated T-cells, we can cite the study from Yang et al., who reported that, in CD3/CD28 pre-primed conditions, IL-6 did not show any significant effect on the CD69 expression in CD8^+^ T-cells isolated from mice [46].

Natural killer cells and CD8^+^ T-cells are both cytotoxic effectors of the immune system and valuable targets for immunotherapies. While NK cells were originally defined as effectors of innate immunity, it is now well accepted that they can influence both innate and adaptive immunity [47]. Regarding the CD8^+^ T-cells, they also play roles in both innate and adaptive immunity [48]. Our data in these two cell populations could finally suggest that the unitary-MI product IL-6 (4 CH) may act as a co-stimulatory signal for T-cells, but especially for NK cells, when combined with the anti-CD3. This body of data provides, for the first time, information about the effect of IL-6 (4 CH) on the activation status of immune cells, which still needs further investigations. Thus, we are aware about the limitations of the study. First of all, the sample size is small; thus we could not apply inferential statistics. Concerning the methods employed to recover the PBMCs, we used Ficoll^®^ gradient, which is not the best method to retrieve the populations of granulocytes. In the future, their recovery through a Percoll^®^ sedimentation gradient protocol will be considered. In addition, it is worth mentioning that this study was also limited by the choice of the markers for cell discrimination and activation. Indeed, regarding the membrane markers used to identify the granulocytes and the monocytes, several other antibodies could have been implemented such as an anti-CD66b or an anti-CD68, for granulocytes and monocytes/macrophages, respectively. Moreover, concerning the choice of the activation markers of interest, our study was solely restricted to the evaluation of the expression of CD69 and HLA-DR, and additional markers such as CD80, CD83, and/or CD86 for the monocyte/macrophage subset or CD38, PD-1, and perforin for the CD8^+^ and the NK cells could have added more robustness to the results. Finally, other fluorometric-based quantification techniques could also have been implemented to confirm our cell count measures, such as carboxy-fluorescein succinimidyl ester (CFSE) dye, that could have easily been added to the panel in this analysis and which will be considered in the future. 

While more studies are needed to better elucidate and confirm these results, these data are quite encouraging for future clinical applications in the context of infectious diseases, immunosurveillance, and cancer immunotherapies [44,49]. 

Not only IL-6 was shown to be involved in cellular activation but also in the determination of the cellular fate: indeed, in serum-containing cultures supplemented with granulocyte macrophage colony-stimulating factor (GM-CSF) + IL-4, IL-6 has been found to be responsible, at least partially, for the differentiation of CD14^+^ monocytes into CD14^+^ CD1a^-^ macrophages [50]. Keeping in mind the interconnection between the innate and the adaptive side of the immunity, and as our results suggest that the unitary-MI product IL-6 (4 CH) could activate, even if just slightly, the monocyte/macrophage sub-population (Figure 1D), we then wanted to check if this MI preparation could have an impact on the polarization state of human macrophages. In particular, we seek to assess the changes induced by IL-6 (4 CH) on the expression of the membrane markers CD14, CD16, CD163, CD200R, and HLA-DR (Figure 3) and on the secretion of a panel of cytokines including TNF-α, IL-1RA, IL-12p40, IL-23, TARC, IP10, IFN-γ, and IL-10 (Figure 4). In our differentiation control macrophages’ model, we found that the M2a phenotype signature corresponded to a decrease in the CD14/CD16 expression, a steady expression of CD163, and an increase in the CD200R/HLA-DR expression, when compared with M0 macrophages (Figure 3B–F). Interestingly, when M0 macrophages were treated with IL-6 (4 CH), the consequent modulation in the expression of these five markers tended to follow the same trend and pattern as observed in the M2a-differentiated macrophages’ sub-type (Figure 3G–K). Additionally, regarding cytokine secretion, M2a macrophages also displayed an increased production of TNF-α, IL-1RA, IL-12p40, IL-23, and IFN-γ, accompanied by a reduced production of IP10 compared with the M0 macrophages (Figure 4B–E,K,L). Interestingly, this secretion profile was also found in the IL-6 (4 CH)-treated macrophages when compared with the Veh. ones (Figure 4F–I,O,P). While it is necessary to consider that macrophages are an extremely plastic cell population and the membrane-bound markers and cytokines assessed here are not sufficient to affirm a bona-fide complete engagement of macrophages into a permanent well-defined sub-type, our results suggest that IL-6 (4 CH) could contribute to the promotion of an M2a-like phenotype. These results could be in accordance with the fact that IL-6 is known to trigger STAT3 signaling [51], and that the latter has been reported to be involved in M2a polarization [52]. Interestingly enough, Fernando et al. have already documented that, when combined with IL-4 and IL-13, IL-6 enhanced the polarization of alternatively activated macrophages, based on their increased expression of arginase-1, Ym1, and CD206 [53]. Moreover, these authors also showed that, when employed alone, IL-6 increased the production of IL-10 and drastically reduced the lipopolysaccharide (LPS)-induced secretion of TARC, which, once again, corroborates our results (Figure 4N,Q). To provide an example of a research study that aimed at investigating the effects of an IL-6 inhibition, we can cite a study supervised in a mice model of spinal cord injury treated with an anti-IL-6R that showed that the inhibition of the IL-6 pathway was associated with a local reduction in the M1 macrophages, found at the lesion site, and an increase in the M2 ones, during the acute phase after injury [54]. However, it is important to remember that macrophage polarization is a flexible process that should be seen as a continuum. Indeed, as these cells can adopt intermediate phenotypes in response to their micro-environment at specific times, their sub-populations can often be very heterogeneous. In addition, the current classification made to distinguish the different macrophage phenotypes could further evolve [55]. Finally, to discuss the overall results of the experiment performed in CD14^+^-derived macrophages, it can be said that IL-6 (4 CH) may have modulated the secretion of both pro-inflammatory and anti-inflammatory makers. These modulatory effects were possibly illustrated by (i) the induction of the expression of pro-inflammatory cytokines such as TNF-α, IL-12p40, IL-23, and IFN-γ, as well as some (ii) anti-inflammatory regulators such as IL-10 and IL1RA, in comparison with the Veh. control. The duality of the effects of IL-6 (4 CH) documented here resonates with the work from Hurst et al., who reported that the sIL-6R/IL-6 complex acted as a switch between the initial neutrophil’s recruitment vs. the mononuclear cell’s infiltration observed during acute inflammation [56]. While the pro-inflammatory role of IL-6 has been the main topic of interest within the current manuscript, due to the role of this cytokine in the acute phase response and in mediating the transition from innate to adaptive immunity during infection or tissue injury, recent evidence has unveiled a more nuanced character for IL-6, highlighting its anti-inflammatory capabilities. In the context of inflammation, IL-6 can indeed act to limit the immune response, thereby conferring a protective effect against excessive damage, a facet of this cytokine which is worth recalling in the present discussion. This anti-inflammatory role is partly mediated through the ability of IL-6 to induce the expression of anti-inflammatory cytokines such as IL-1Ra [57] and IL-10 [58]. Such a dichotomic effect is important to mention in the context of our study as we rightly highlighted here the potential effect of the MI preparation of IL-6 (4 CH) in slightly enhancing the secretion of IL-1Ra and IL-10 (Figure 4G,Q). Of significant interest is the dual-functionality model of IL-6, whereby its activity is context-dependent: for example, in one scenario, it promotes inflammation by supporting the differentiation of Th17 cells, while in another, it fosters the expansion of regulatory T-cells (Tregs), which are pivotal in resolving inflammation and maintaining immune homeostasis [59]. Transposed to our results, these anti-inflammatory facets of IL-6 could possibly explain the fact that IL-6 (4 CH) has favored a M2a-macrophage polarization profile (Figure 2 and Figure 3) [60]. As the balance between these facets of IL-6 is thought to be critical in the pathogenesis and resolution of various inflammatory diseases, this study, once again, illustrates that the overall biological responses should be seen as a continuum. Based on this example and the well-known pleiotropic role of IL-6, our results are finally not surprising and lay the foundations for further studies to explore their potential uses in clinics. 

### 4.3. Discussion about the Effects of Ultra-Low Doses of IL-6, Studied through One Capsule of the Complex-MIM 2LALERG^®^

In the second part of the current study, we wanted to assess the anti-inflammatory effect of IL-6, when used at ultra-low doses (ULD) and combined with other active ingredients into the complex-MIM 2LALERG^®^. The effects of this complex-MIM was evaluated in a double-blind placebo-controlled clinical study that showed encouraging results about the efficacy of the medicine in reducing rescue medicines uptake in patients suffering from seasonal allergic rhinitis [61]. In addition to this clinical study, a preclinical in vivo study implemented other evidence supporting the potential of the medicine in the context of IgE-mediated inflammation. Indeed, this medicine, administered sequentially, according to the human posology, in a murine model of pollen-induced allergic inflammation, reduced the mucus accumulation within the lungs and decreased the number of eosinophils, as well as the levels of IL-4, IL-5, and IL-13 found in bronchoalveolar lavage fluids [28]. Macrophages are abundant in the lungs (about 70% of the immune cell populations) and play a critical role in maintaining the immune responses in the context of respiratory inflammation [62]. Considering the possible effects of IL-6 (4 CH) obtained on those cells (Figure 1C,D, Figure 3, and Figure 4), the second aim of the current study was to complement this first set of in vivo results, putting more emphasis on macrophages. In the context of chronic inflammation-mediated diseases, and more specifically, in allergic diseases, macrophages play a dual role, both in (i) helping the resolution of inflammation and (ii) sustaining the progression of the inflammatory-mediated disease [55]. For this reason, we believe that assessing the effects on this target cell population is very important. 

Interestingly, our results showed that the tested capsule of 2LALERG^®^ may have reduced the LPS-induced IL-6 secretion of CD14^+^-derived macrophages, in comparison with the Veh., in a time-dependent manner, the strongest inhibition being observed after 48 h of the 2LALERG^®^ treatment compared with the 24 h one (Figure 5B,D). Regarding the complex nature of the 2LALERG^®^ formulation, and being aware that the tested capsule also contains other actives in association with IL-6 (17 CH), it is possible that the ULD-IL-6 could have played a role in the observed IL-6-down-regulation. Although the mode of action of ULD still needs to be elucidated, these data could finally be put in parallel with the results from four other studies, which have demonstrated that pro-inflammatory cytokines such as IL-1β and TNF-α, either used at 17 CH or at 27 CH in unitary-MI products and/or in complex MIMs, displayed anti-inflammatory properties and the capacity to down-regulate their own secretion and expression [23,24,25,29].

Regarding the results we presented here, they can be viewed as quite interesting since, in the context of allergies, inhibiting IL-6 levels is presumed to be advantageous. This was supported by Neveu et al., who observed significantly higher IL-6 levels in the blood of allergic asthmatic patients compared to healthy individuals [12]. The modalities by which cytokines such as IL-6 are released from a cell and can then mediate signalization within the same cell are not fully understood, especially within macrophages. Kawano et al. were able to detect IL-6 production in freshly explanted human myelomas, and by using antibodies against IL-6, to specifically inhibit its pathway, they found a reduction in cellular proliferation, suggesting an autocrine requirement for this cytokine in this cell type for sustaining its proliferation [63]. Moreover, the evidence of an IL-6-mediated-autocrine growth loop was then confirmed in the human myeloma cells U266 [64]. Nonetheless, while these first results were solely collected from in vitro experiments, previous in vivo results with the 2LALERG^®^ therapy were also able to highlight its systemic effects in reducing pro-inflammatory markers such as IL-4 and IL-5, the latter being employed at ULD in the complete formulation of 2LALERG^®^ [28,33]. Furthermore, as the pharmacological inhibition of IL-6 together with TNF has been suggested to be beneficial for patients suffering from severe allergic asthma [65], it is also interesting to mention that the complete formulation of 2LALERG^®^ also encompasses ULD of TNF-α. Given this, and although these results should also be validated in patients, therapy with 2LALERG^®^ holds promise in the context of allergies. 

Finally, as histamine is a well-known mediator of allergic reactions, the capacity of IL-6 to stimulate mast cells’ histamine degranulation has been documented, and it is even one of the pillars of the rationale for the employment of this cytokine at ULD in the formulation of the MIM 2LALERG^®^ [33]. The final part of the current study thus aimed at assessing the effect of the tested 2LALERG^®^ capsule on the histamine degranulation of rat mast cells. Regarding the experimental protocol followed here, we found that the tested capsule slightly reduced the degranulation of histamine when compared with the Veh., especially after short incubation periods of 15 min (Figure 6B). This fast response is quite impressive and could reveal that an incubation period of only a few minutes with the tested MIM is sufficient to drive cellular responses. Interestingly, our results could be related to the ones from Desai et al., who also reported that IL-6 enhanced human mast cells streptavidin-induced degranulation of β-hexosaminidase [66]. Based on the modulatory/inhibitory principle of ULD employment in MI, the reduced degranulation observed here could be attributable to the combination of the ULD of IL-6 plus the other immune-based actives associated with the tested capsule of 2LALERG^®^. In particular, the presence of pulmo-histaminum at 15 CH could have played a role, too. Indeed, a multi-site study demonstrated the effects of high dilutions of histamine in reducing human basophil activation [67]. 

Regarding the conclusion that can be drawn from the results obtained from our peritoneal mast cells experiment (Figure 6), we are perfectly aware that the choice of a murine model has limitations and that we should be cautious in the extrapolation of these data to humans. Indeed, even if the fundamental organization of the immune system is largely conserved between mice and humans, their immune responses can differ in several key aspects, including the prevalence and function of certain immune cells, the expression and affinity of immune receptors, and the cascade of signaling events following immune activation [68]. Specifically, different relative propensities for developing inflammatory/autoimmune diseases and altered responses to inflammation and infection have even already been reported between rat strains [69].

In spite of the above-mentioned limitation, this final experiment is relevant if we consider the crucial roles played by mast cells in allergic responses and in the defense against pathogens [70]. The observed reduction in histamine release from rat peritoneal mast cells (Figure 6B,D) provides insights into potential pathways that can be targeted for therapeutic interventions, while still being cautious regarding the translation of these findings to humans. Consequently, although a compound that reduces histamine release in rat mast cells is promising, its efficacy and safety in humans must be rigorously tested through clinical trials to account for these differences. Moreover, the systemic effects of such a compound need to be evaluated in the context of the complex human immune network to ensure that beneficial outcomes in rat models translate into therapeutic potential for human conditions such as allergies, asthma, or anaphylaxis.

Concerning the effects of other MIMs that include ULD of IL-6 amongst other active ingredients, their anti-inflammatory properties have already been delineated in previous publications. In particular, these capabilities were evidenced in vitro in LPS-inflamed human primary monocytes and LPS-inflamed human primary granulocytes, as well as in LPS/IFN-γ-inflamed rat cortical neurons and glial cells [32]. 

According to the current knowledge, targeting IL-6 in the context of allergic diseases could be beneficial for patients suffering from atopic dermatitis. Indeed, the inhibition of IL-6R/sIL-6R through the use of the mAb tocilizumab at the dose of 8 mg/kg of body weight every 4 weeks was shown to decrease the clinical signs of atopic dermatitis. However, this treatment was also associated with bacterial superinfections [71], probably because, while IL-6 is important in host immune defenses, its down-regulation affects the capacity of the body to fight against infections. On this subject, finding the right balance in the levels of IL-6 inhibition is of paramount importance for the success of the therapeutic management of allergies, and its use at LD and ULD could be an effective and safe solution. 

Altogether, the data presented in this study could highlight how LD and ULD of IL-6, when included in MIM could either lead to immune cells activation or inhibition in in vitro inflammation-related models.

## 5. Conclusions

In conclusion, the current study aimed at evaluating the immune-modulatory effects of two IL-6-containing micro-immunotherapy (MI) formulations in an in vitro study of (i) the unitary product IL-6 (4 CH) and (ii) one capsule of the complex-MIM 2LALERG^®^, which encompasses IL-6 (17 CH) in its formulation, in human peripheral blood mononuclear cells (PBMCs)-derived cells and mast cells retrieved from rats. From a fundamental perspective, our results seemed to show that, under basal conditions, IL-6 (4 CH) was able to activate granulocytes and, to a lesser extent, monocytes/macrophages. Moreover, in a CD3-primed state, a different activation pattern was possibly highlighted, as IL-6 (4 CH) was more prone to activate natural killer (NK) cells, as well as CD8^+^ T-cells, the latter being to a smaller extent. In addition, as suggested by its effects on the expression of some membrane-bound markers and cytokine secretion profile, it seemed that IL-6 (4 CH) could also possibly sustain the M2a-macrophage differentiation’s signature, at least characterized by reduced levels of CD14, CD16, and IP10 and increased levels of CD200R, HLA-DR, TNF-α, IL-1Ra, IL-12p40, and IL-23. Altogether, and even if relatively preliminary, these results may suggest that IL-6, when employed at a low dose (LD), could activate some functional properties, such as cell proliferation and immune cell activation, thus acting as an immunostimulant, as it has previously been shown for LD of IFN-γ [30]. On the other hand, the anti-inflammatory potential of IL-6 (17 CH), when included in the 2LALERG^®^, has also been addressed. Indeed, the tested capsule of this medicine seemed to reduce the release of IL-6 itself in lipopolysaccharide (LPS)-inflamed human macrophages, and it also inhibited the histamine degranulation of rats’ peritoneal mast cells. These additional data, even if they still need to be completed, definitely contribute to adding more information about ULD-IL-6 when employed in MI, thus strengthening the current knowledge about the mode of action of 2LALERG^®^, previously reported in an in vivo model of allergic disease [28,33]. To conclude, the overall study, by addressing both sides of the LD/ultra-low dose (ULD) ranges used in MI, provided novel pieces of evidence on their double-edged potential in modulating the immune responses. 

## Data Availability

The data of the current study are available from the corresponding author upon reasonable request.

## Supplementary Materials

The following supporting information can be downloaded at: https://www.mdpi.com/article/10.3390/life14030375/s1, Supplementary Figure S1: Representative scheme of the IL-6-targeting strategy employed in micro-immunotherapy (MI), and overview of the study. Supplementary Figure S2: Granulocytes are activated by concanavalin A. Supplementary Figure S3: Natural killer cells and CD8^+^ T-cells are activated by concanavalin A and by anti-CD3 signal. Supplementary Figure S4: The unitary micro-immunotherapy (MI) product IL-6 (4 CH) does not impact the cell viability of the CD14^+^-derived macrophages in control conditions. Supplementary Figure S5: The unitary micro-immunotherapy (MI) product IL-6 (4 CH) does not impact the cell viability of the CD14^+^-derived macrophages in the presence of lipopolysaccharide (LPS). Table S1: Summary of the mean cell count amongst the three assessed donors (#A, #B, and #C), in basal culture condition, per analyzed peripheral blood mononuclear cells (PBMCs) in the cell sub-populations of interest, in vehicle- (Veh.) and in IL-6 (4 CH)-treatment conditions., Table S2: Summary of the mean cell count amongst the three assessed donors (#A, #B, and #C), in the presence of an anti-CD3 in the culture medium, per analyzed peripheral blood mononuclear cells (PBMCs) in the cell sub-populations of interest, in vehicle- (Veh.) and in IL-6 (4 CH)-treatment conditions..

## Abbreviations

Ab: antibody; ADAM17: a disintegrin and metalloprotease 17; BSA: bovine serum albumin; CH: centesimal Hahnemannian; Con A: concanavalin A; Crgly: cromoglycate; DNA: deoxyribonucleic acid; EBSS: Earle’s balanced salt solution; EIA: enzyme immunoassay; ELISA: enzyme-linked immunosorbent assay; FBS: fetal bovine serum; FCS: fetal calf serum; GM-CSF: granulocyte macrophage colony-stimulating factor; gp130: glycoprotein receptor 130 kDa; HEPES: 4-(2-hydroxyethyl)-1-piperazineethanesulphonic acid; HLA: human leukocyte antigen; hr: human recombinant; IFN: interferon; Ig: immunoglobulin; IL: interleukin; IL-1RA: interleukin-1 receptor antagonist; IL-6: Interleukin-6; IL-6R: IL-6 receptor; IP10: interferon gamma-induced protein 10; JAK: Janus kinase; LDs: low doses; LPS: lipopolysaccharide; M-CSF: macrophage colony-stimulating factor; MEM: minimum essential media; MI: micro-immunotherapy; MIM: micro-immunotherapy medicine; NK: natural killer; OD: optical density; PBMCs: peripheral blood mononuclear cells; PBS: phosphate buffer saline; P/S: penicillin/streptomycin; RNA: ribonucleic acid; RPMI: Roswell Park Memorial Institute; RT: room temperature; S.D.: standard deviation; S.E.M.: standard error of the mean; sIL-6R: soluble IL-6 receptor; SN: supernatant; STAT: signal transducer and activator of transcription; TARC: thymus and activation-regulated chemokine; TGF-β: transforming growth factor-β; Th2: T helper cell type 2; TNF-α: tumor necrosis factor-α; ULD: ultra-low doses; Veh.: vehicle.
