# Supplementary material for: In Vitro Study of Interleukin-6 when Used at Low Dose and Ultra-Low Dose in Micro-Immunotherapy
Source: Life (Basel). 2024 Mar 12;14(3):375. doi: 10.3390/life14030375 (PMC10971188; doi:10.3390/life14030375)
Supplement: Supplementary file 1 [file life-14-00375-s001.zip › life-2893795-supplementary.pdf]

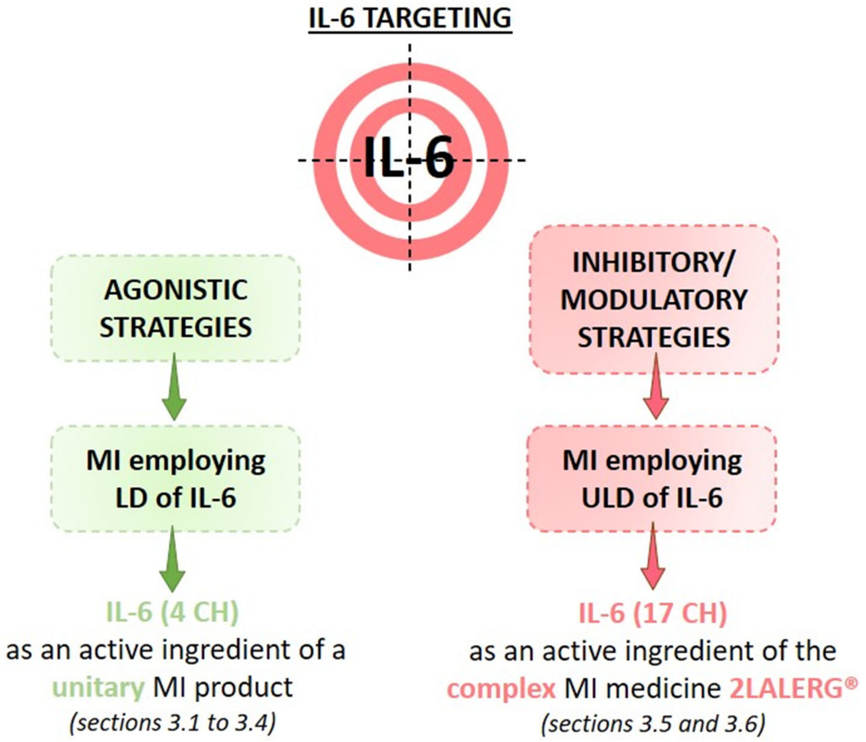

Supplementary Figure S1: Representative scheme of the IL-6-targeting strategy employed in micro-immunotherapy (MI), and overview of the study. The left panel (green boxes) depicts the agonistic strategy employed by MI, through the use of low doses (LD) of IL-6. The effects of the unitary-MI product IL-6 (4 CH) are further reported in sections 3.1 to 3.4 of the current manuscript. The right panel (red boxes) illustrates the modulatory/inhibitory strategy employed by MI, through the use of ultra-low doses (ULD) of IL-6. Thus, the assessment of the effects of one capsule belonging to the complex micro-immunotherapy medicine (MIM) 2LALERG®, and employing IL-6 at (17 CH), is evaluated in sections 3.5 and 3.6 of the current manuscript. CH: centesimal Hahnemannian; LD: low doses; MI: micro-immunotherapy; MIM: micro-immunotherapy medicine; ULD: ultra-low doses.

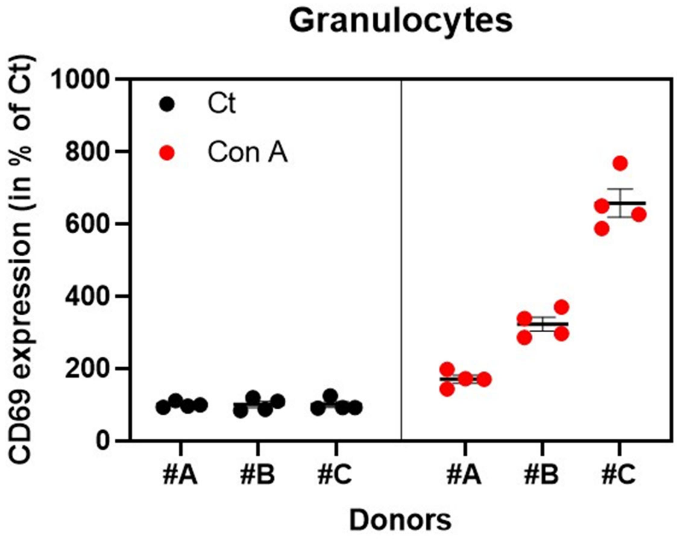

Supplementary Figure S2: Granulocytes are activated by concanavalin A. The validity of the activations' capabilities of granulocytes was assessed by measurement of the CD69 expression levels via flow cytometry, after having incubated the cells for 48 hours alone (Ct) or in the presence of 5 μg/mL concanavalin A (Con A). The activation status of granulocytes has been evaluated in peripheral blood mononuclear cells (PBMCs) isolated from three healthy donors (#A, #B, and #C). For each donor, the data are presented as the mean ± standard error of the mean (S.E.M.) results obtained for *n* = four replicates for each donor and is expressed as a percentage of the Ct (set at 100% for each donor). Each black dot represents one replicate per donor in the Ct condition, whereas red dots represent the replicates in the Con A condition.

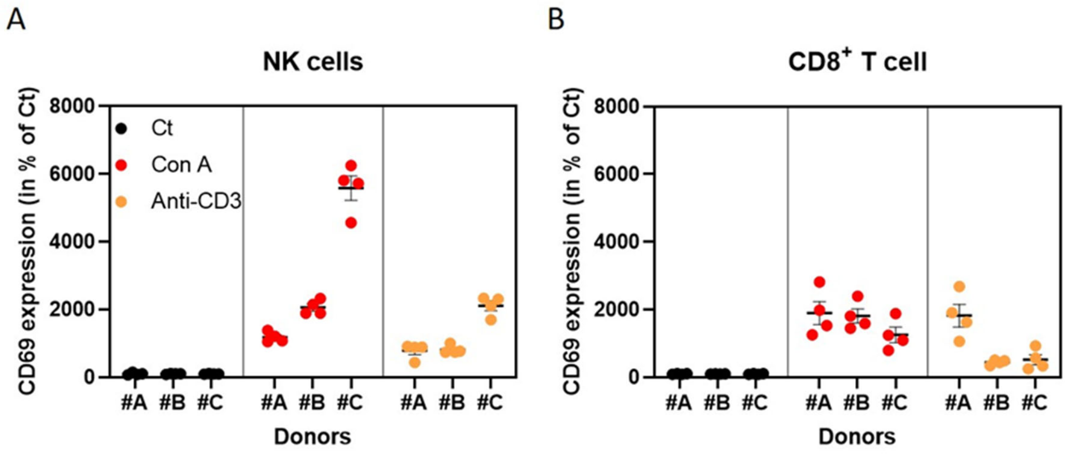

Supplementary Figure S3: Natural killer cells and CD8+ T-cells are activated by concanavalin A and by anti-CD3 signal. The validity of the activations' capabilities of the natural killer (NK) cells and the CD8+ T-cells were assessed by measurement of the CD69 expression levels via flow cytometry, after having incubated the cells for 48 hours alone (Ct) in the presence of 5 µg/mL concanavalin A (Con A) or in the presence of 0.5 µg/mL anti-CD3 (anti-CD3). The activation status of NK cells (**A**) and CD8+ T-cells (**B**) were evaluated in peripheral blood mononuclear cells (PBMCs) isolated from three healthy donors (#A, #B, and #C). For each donor, the data are presented as the mean ± standard error of the mean (S.E.M.) results obtained for *n* = four replicates for each donor and is expressed as a percentage of the Ct (set at 100% for each donor). Each black dot represents one replicate per donor in the Ct condition, red dots represent the replicates in the Con A condition, whereas orange dots represent the replicates in the anti-CD3-treated condition.

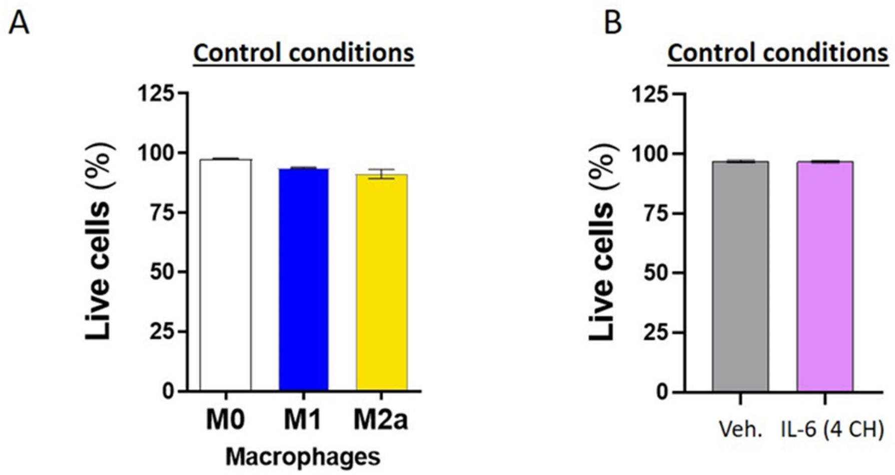

Supplementary Figure S4: The unitary micro-immunotherapy (MI) product IL-6 (4 CH) does not impact the cell viability of the CD14$^+$-derived macrophages, in control conditions. The viability of the macrophages was assessed via flow cytometry in control conditions, after a 7-day culture of the CD14$^+$-derived macrophages either in complete medium alone (M0), ± 50 ng/mL IFN-γ (M1), ± 50 ng/mL IL-4 (M2a), ± 50 ng/mL IL-10 (M2c) (**A**); or ± vehicle (Veh.), ± IL-6 (4 CH) (**B**). The results are expressed in % of live cells amongst the number of total cells analyzed for each condition, based on the gating of the NIR-Zombie-positive cells.

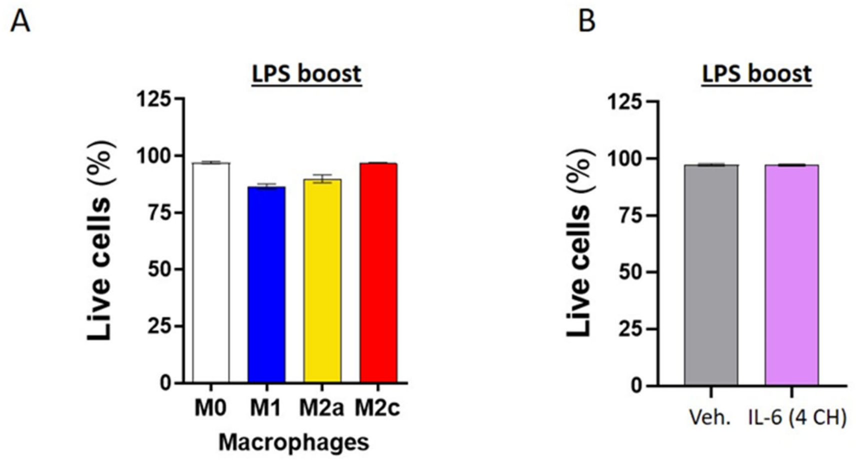

Supplementary Figure S5: The unitary micro-immunotherapy (MI) product IL-6 (4 CH) does not impact the cell viability of the CD14^{+}-derived macrophages in the presence of lipopolysaccharide (LPS). The viability of the macrophages was assessed via flow cytometry in the presence of a 100 ng/mL LPS boost, after a 7-day culture of the CD14^{+}-derived macrophages either in complete medium alone (M0), ± 50 ng/mL IFN-γ (M1), ± 50 ng/mL IL-4 (M2a), ± 50 ng/mL IL-10 (M2c) (**A**); or ± vehicle (Veh.), ± IL-6 (4 CH) (**B**). The results are expressed in % of live cells amongst the number of total cells analyzed for each condition, based on the gating of the NIR-Zombie-positive cells.

Supplementary Table 1. Summary of the mean cell count amongst the three assessed donors (#A, #B, and #C), in basal culture condition, per analyzed peripheral blood mononuclear cells (PBMCs) in the cell sub-populations of interest in vehicle- (Veh.) and in IL-6 (4 CH) treatment conditions.

	**Cell count amongst *n* = three donors**	
	Mean percentage ± S.D. (Veh. as 100%)	
**Basal culture condition**	**Veh.**	**IL-6 (4 CH)**
**Granulocytes**	100.00	196.84 (± 19.62)
**Monocytes/macrophages**	100.00	47.59 (± 8.02)

IL: interleukin; S.D.: standard deviation of the mean; Veh.: vehicle.

Supplementary Table 2. Summary of the mean cell count amongst the three assessed donors (#A, #B, and #C) in the presence of an anti-CD3 in the culture medium, per analyzed peripheral blood mononuclear cells (PBMCs) in the cell sub-populations of interest, in vehicle- (Veh.) and in IL-6 (4 CH) treatment conditions.

	**Cell count amongst *n* = three donors**	
	Mean percentage ± S.D. (Veh. as 100%)	
**Anti-CD3 condition**	**Veh.**	**IL-6 (4 CH)**
**NK cells**	100.00	82.08 (± 8.11)
**$CD8^+$ T-cells**	100.00	77.67 (± 4.11)

IL: interleukin; NK: natural killer; S.D.: standard deviation of the mean; Veh.: vehicle.
